# Supplementary material for: Copy number rather than epigenetic alterations are the major dictator of imprinted methylation in tumors
Source: Nat Commun. 2017 Sep 7;8:467. doi: 10.1038/s41467-017-00639-9 (PMC5589900; doi:10.1038/s41467-017-00639-9)
Supplement: Supplementary file 1 — Supplementary Information [file 41467_2017_639_MOESM1_ESM.pdf]

Description of Supplementary Files

File Name: Supplementary Information
Description: Supplementary Figures

File Name: Supplementary Data 1
Description: Calculated basal ploidy for the cancer cell lines used in this study.

File Name: Supplementary Data 2
Description: HM450k probes mapping to imprinted DMRs in control matched tissues, cancer cell lines, TCGA primary tissue datasets.

File Name: Supplementary Data 3
Description: Extent of internal and telomere/centromere CNAs encompassing imprinted loci for all cells lines

File Name: Supplementary Data 4
Description: The frequency of CNAs encompassing both imprinted loci and nearby tumour-supressor and oncogenes

File Name: Supplementary Data 5
Description: The extent of imprint-encompassing cnnLOH in cancer cell lines and accompanying homozygous mutated genes

File Name: Supplementary Data 6
Description: The average methylation β values for Illumina Infinium HM450k probes mapping to CIMP, BIV and DMV in cancer cell lines

File Name: Supplementary Data 7
Description: Details of the TCGA characteristics including estimates (as %) for necrosis, immune cell infiltrate, stromal content and tumour mass.

File Name: Supplementary Data 8
Description: The average methylation β values for Illumina Infinium HM450k probes mapping to CIMP, BIV and DMV in primary TCGA tissues

File Name: Supplementary Data 9
Description: Extracted methylation values for all HM450k probes mapping to the IGF2-H19 DMRs in paired TCGA primary tumours and normal adjacent tissue

File Name: Supplementary Data 10
Description: PCR primer sequences used in this study

File Name: Peer Review File

Supplementary Figure 1

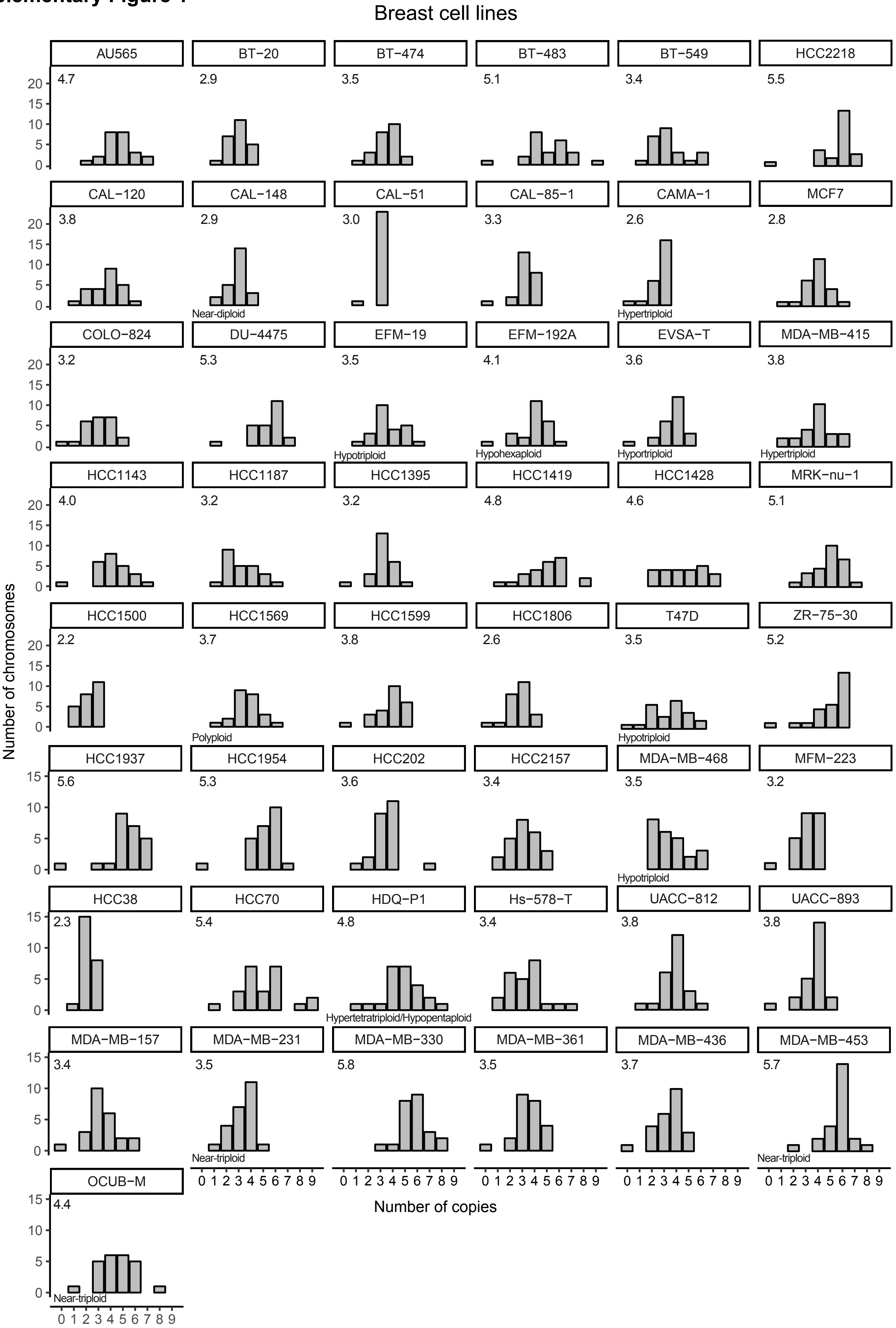

Supplementary Fig. 1. Bar graphs depicting the distribution of chromosomes and ploidy status for breast cell lines used in this study. When reported, the supplier's ploidy definition is given.

Supplementary Figure 2

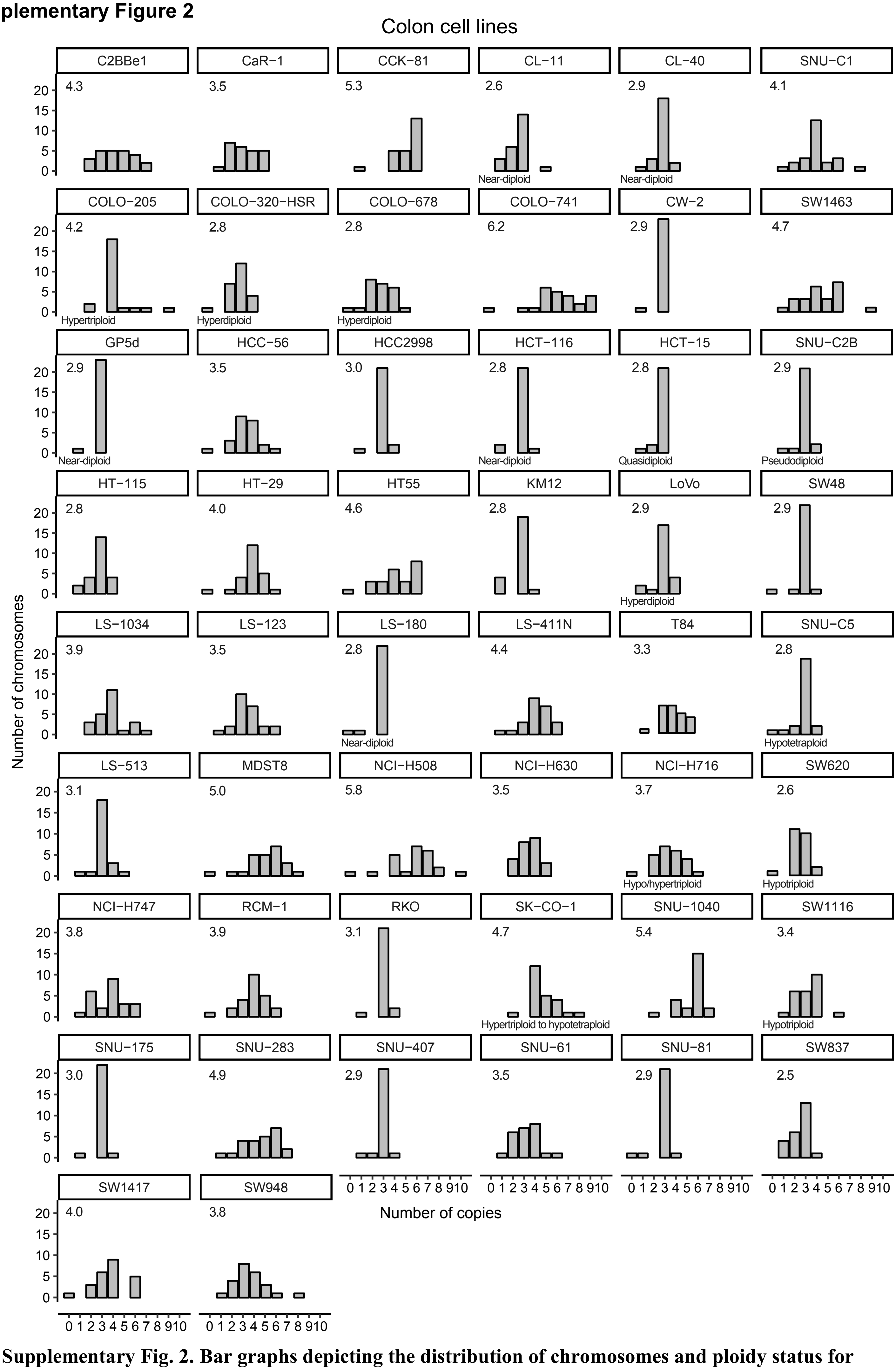

Supplementary Fig. 2. Bar graphs depicting the distribution of chromosomes and ploidy status for colon cell lines used in this study. When reported, the supplier's ploidy definition is given.

Supplementary Figure 3

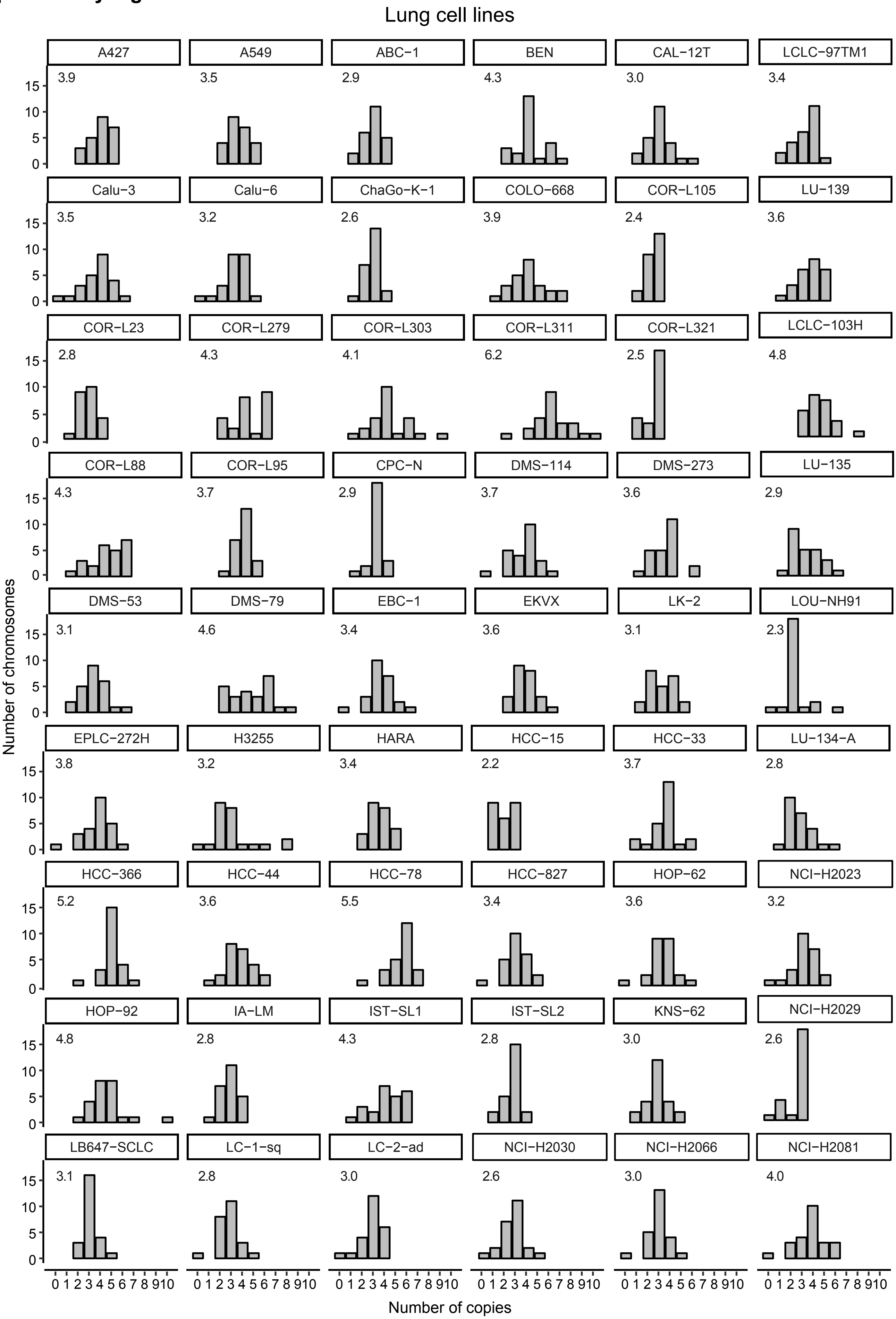

Supplementary Fig. 3. Bar graphs depicting the distribution of chromosomes and ploidy status for lung cell lines used in this study. When reported, the supplier's ploidy definition is given.

Supplementary Figure 4

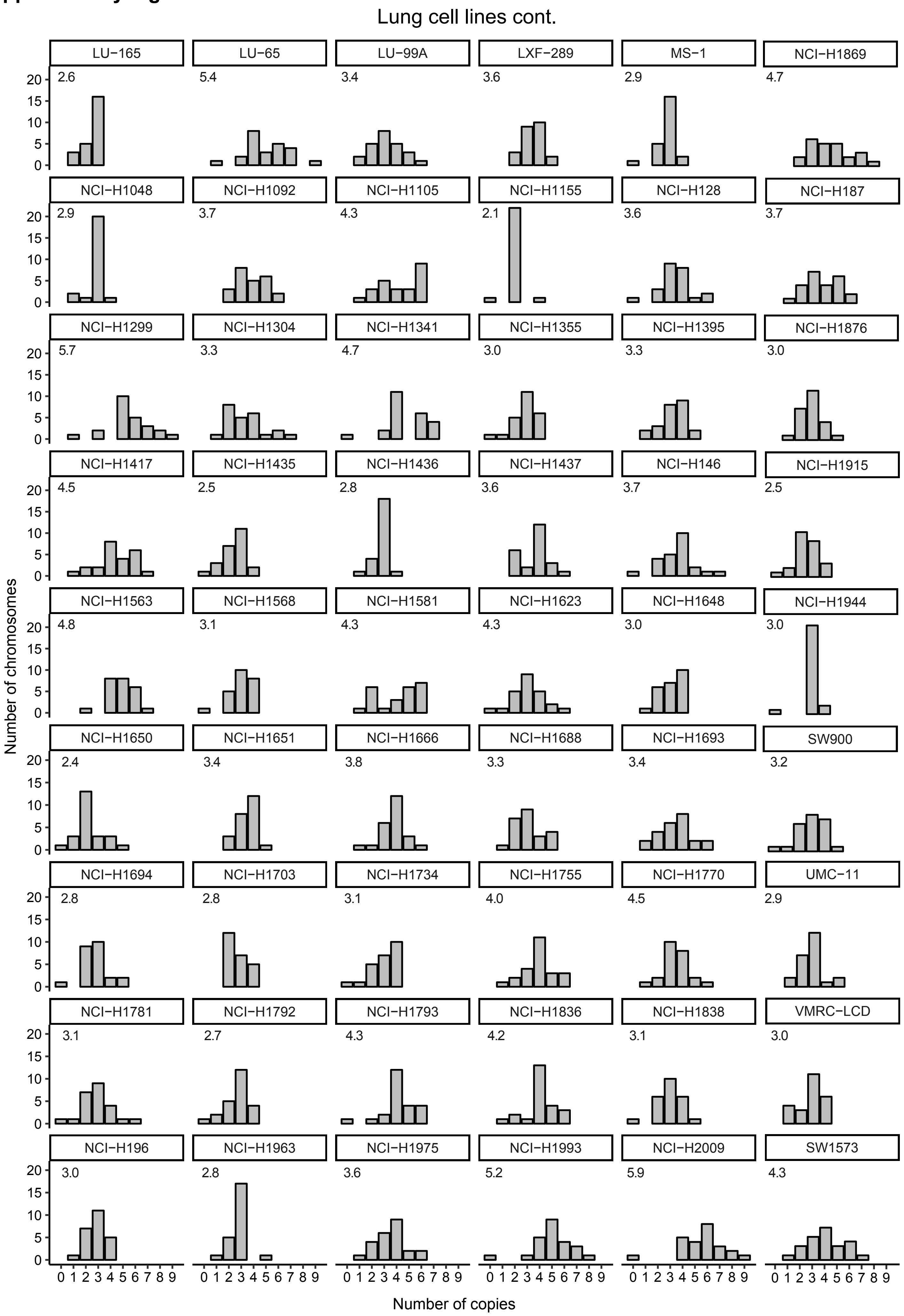

Supplementary Fig. 4. Bar graphs depicting the distribution of chromosomes and ploidy status for lung cell lines used in this study. When reported, the supplier's ploidy definition is given.

Supplementary Figure 5

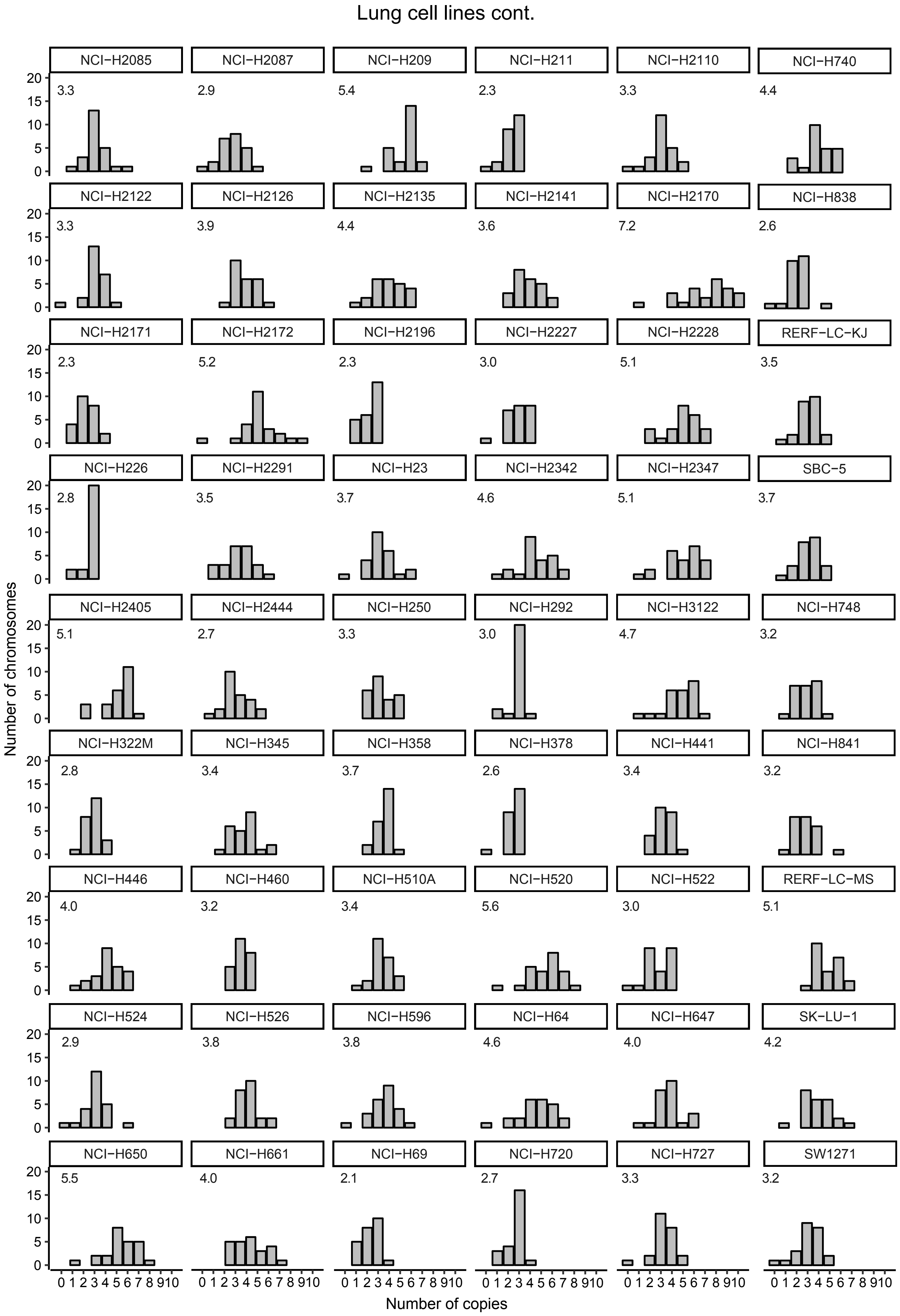

Supplementary Fig. 5. Bar graphs depicting the distribution of chromosomes and ploidy status for lung cell lines used in this study. When reported, the supplier's ploidy definition is given.

Supplementary Figure 6

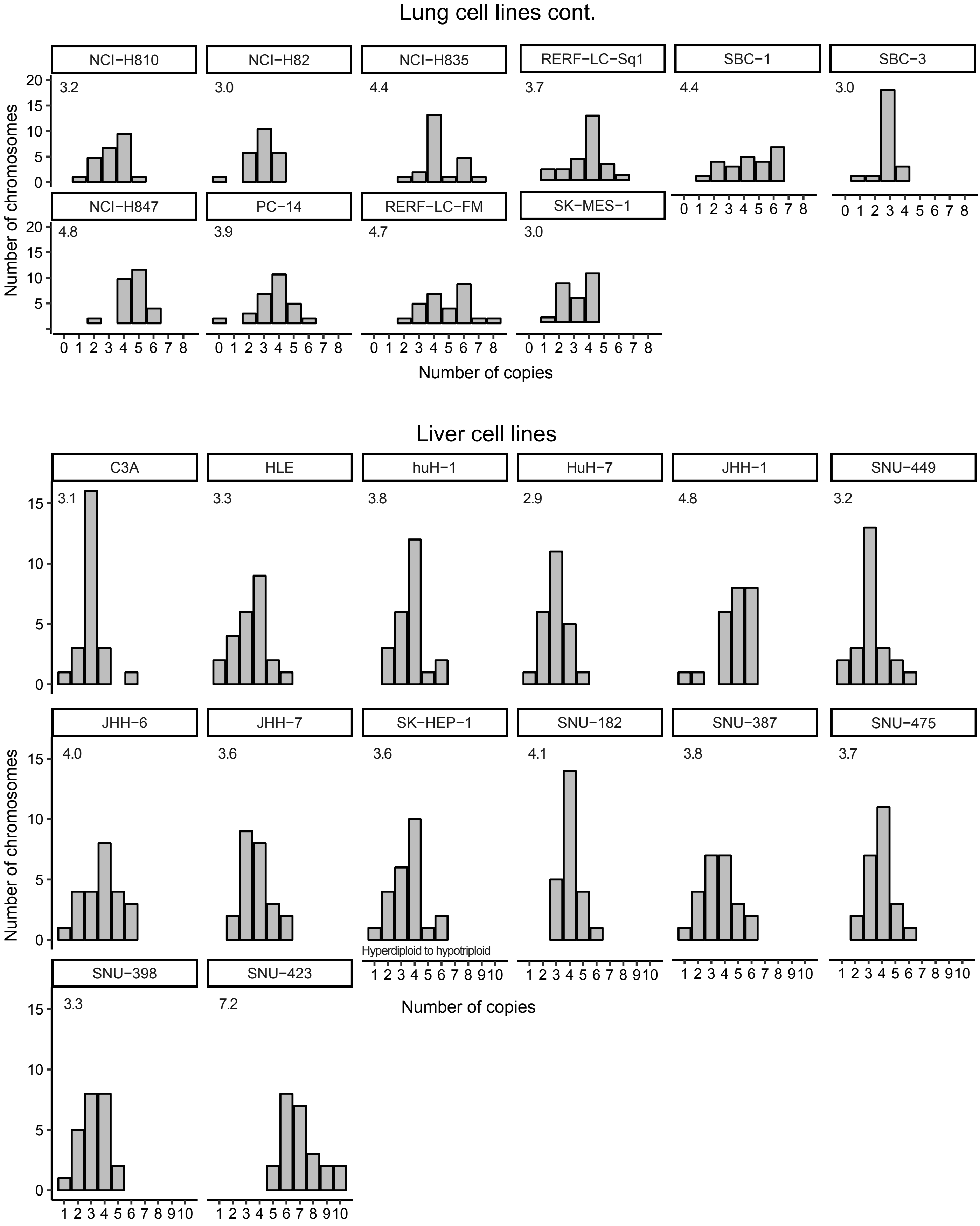

Supplementary Fig. 6. Bar graphs depicting the distribution of chromosomes and ploidy status for lung and liver cell lines used in this study. When reported, the supplier's ploidy definition is given.

Supplementary Figure 7

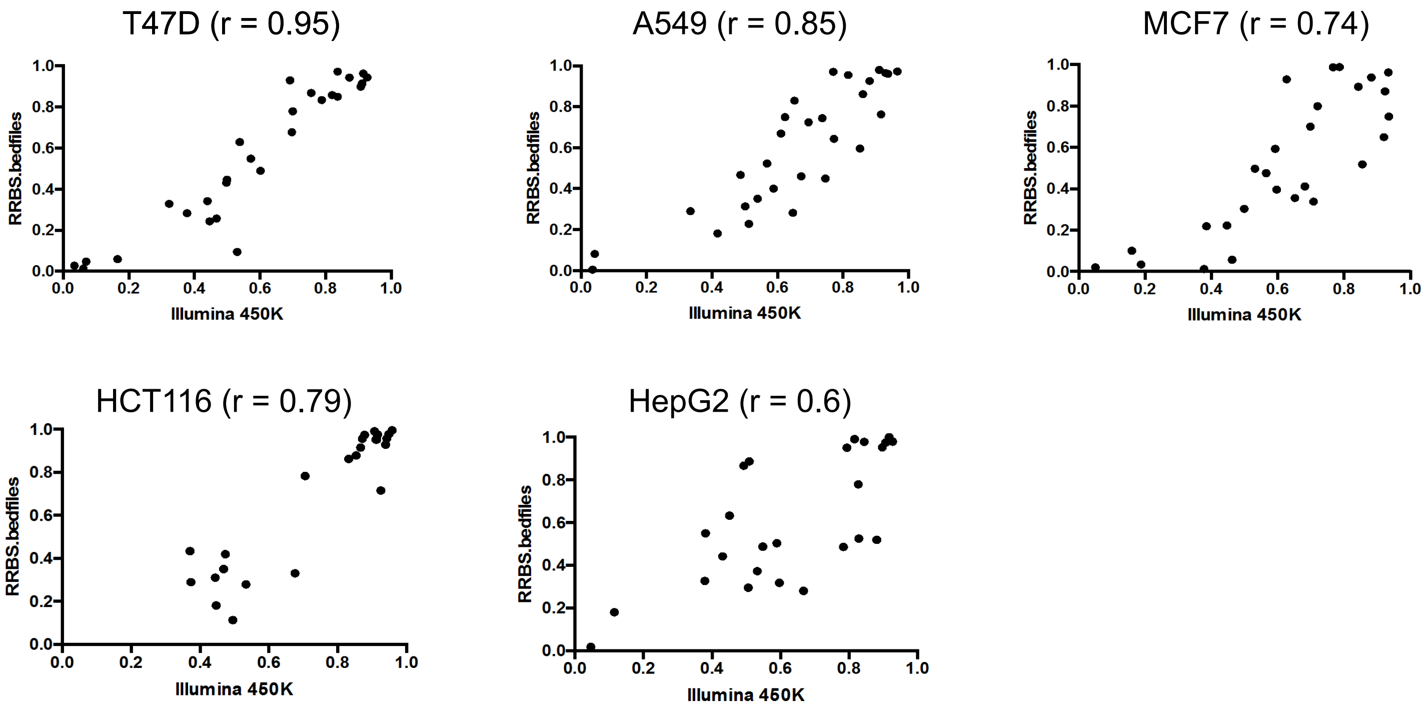

Supplementary Fig. 7. Data plots for methylation of CpG dinucleotides assessed by both HM450k array and ENCODE RRBS for five cancer cell lines. Each dot represents a single CpG data point.

Supplementary Figure 8

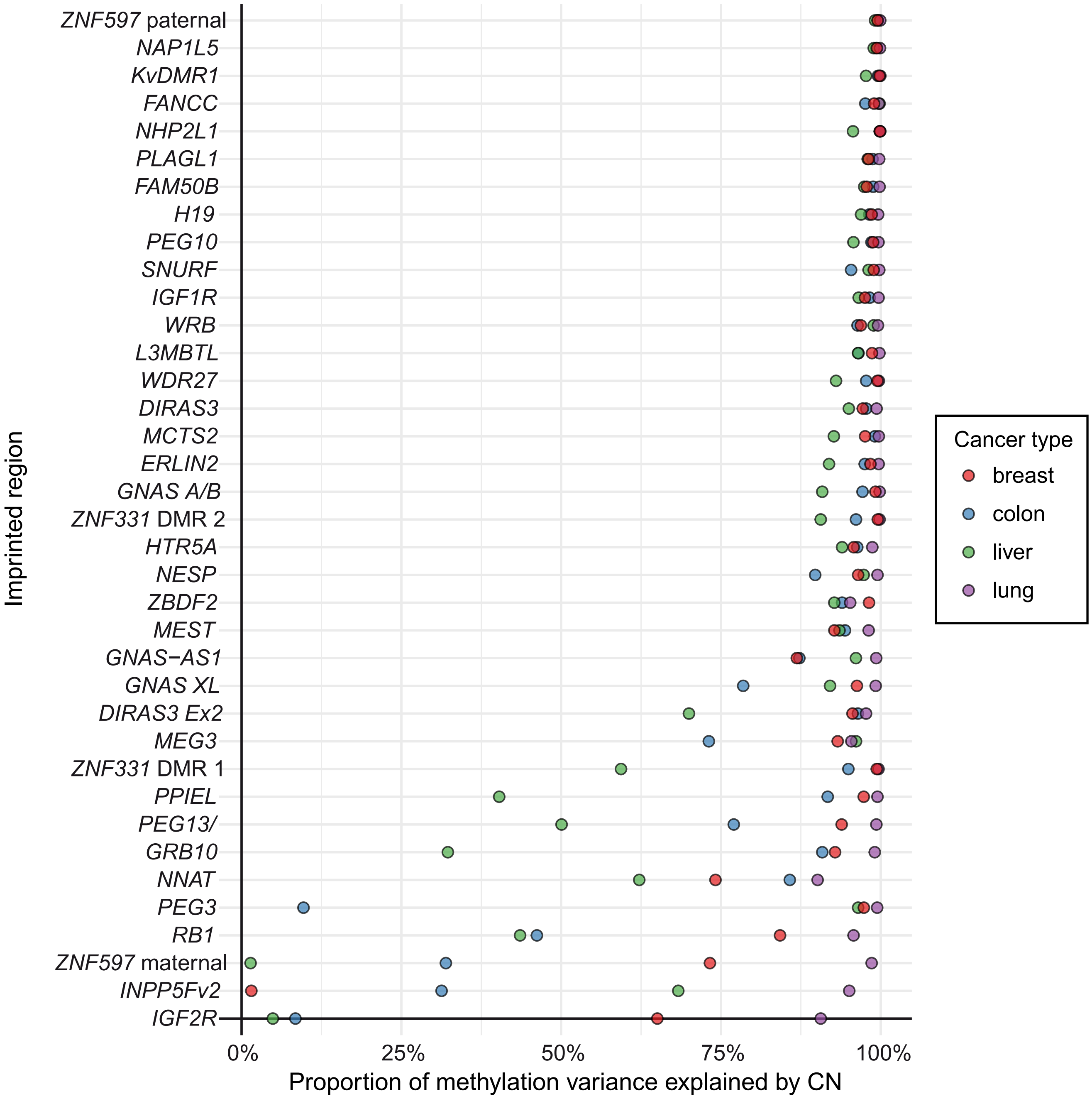

Supplementary Fig. 8. Graphical representation of the proportion of methylation variance explained by copy-number for all cancer cell lines. Imprinted DMRs are arranged in descending ordered according to the degree by which copy-number explains the largest proportion of methylation vaiance.

Supplementary Figure 9

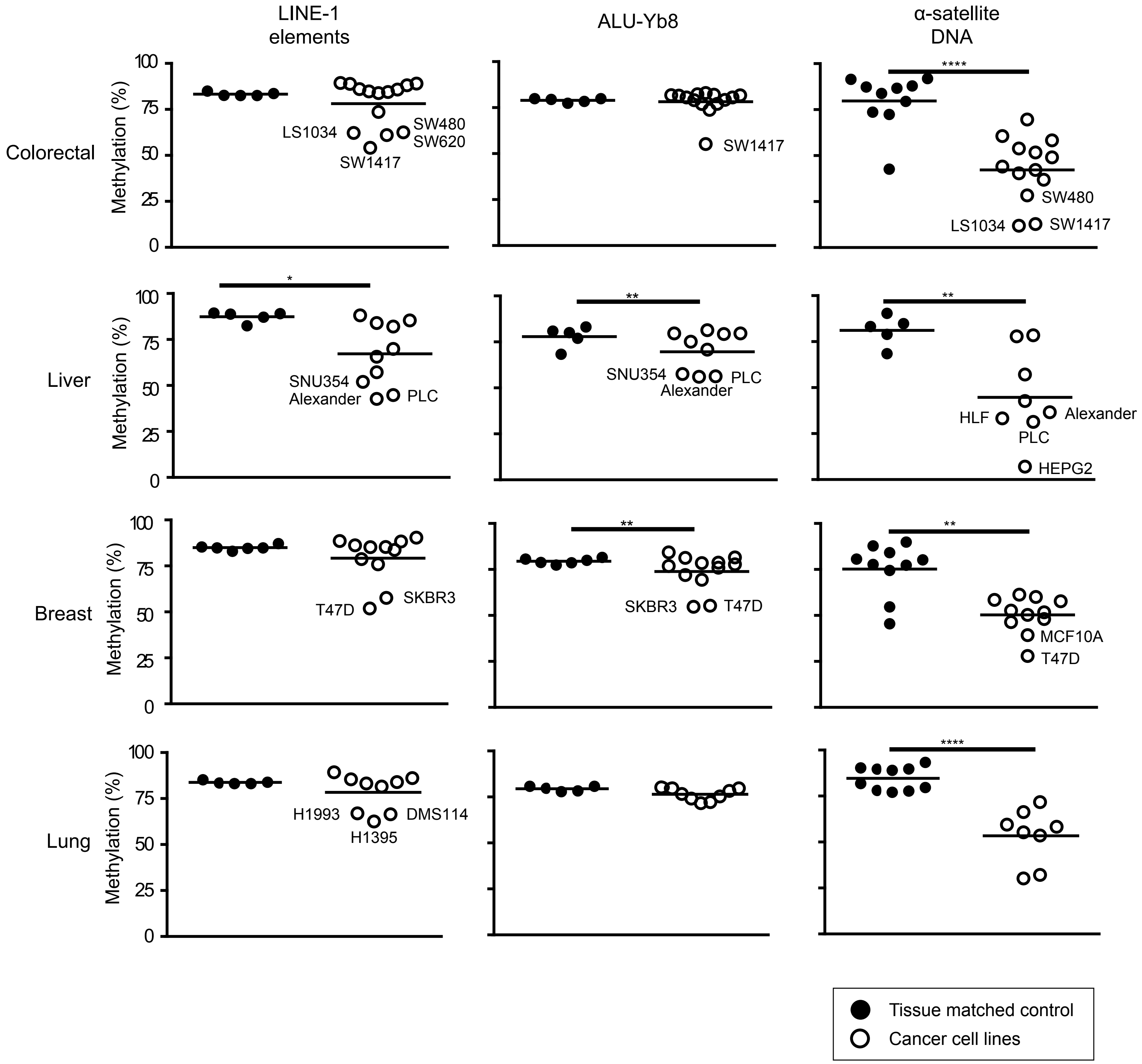

Supplementary Fig. 9. Quantification of methylation at LINE-1, ALU-Yb8 and α-satellites using pyrosequencing. The methylation profiles of cancer cell lines were compared to normal tissue matched controls. Cell lines with robust hypomethylated are identified.

Supplementary Figure 10

NAP1L5

Tissue	Cancer Cell line	*NAP1L5* Methylation (%)	CNV (total:minor allele)	rs710834 gDNA	rs710834 cDNA
Breast	BT-474	56	2:1	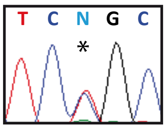	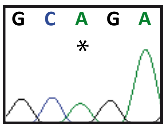
	HCC38	55	2:1	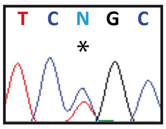	No expression
	MCF7	66	3:1	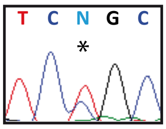	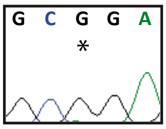
	MDA-MB-453	57	4:2	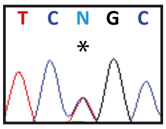	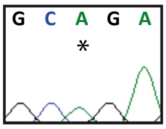
Colon	Gp5D	50	2:1	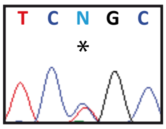	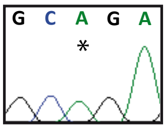
	HCT-116	92	2:1	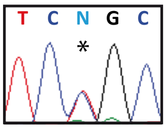	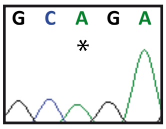
	HCT-15	53	2:1	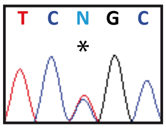	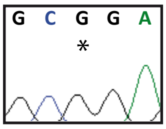
	LoVo	64	2:1	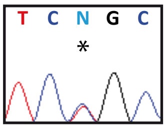	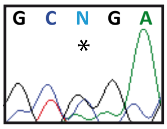
	COLO-205	63	3:1	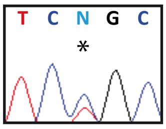	No expression

Supplementary Fig. 10. Allelic expression analysis of 10 imprinted transcripts in cancer cell lines. The CNA status and average methylation for each DMR is shown alongside Sanger sequencing traces of highly informative polymorphisms in the *NAPL1L5* transcript which were used for qualitative allelic expression analyses.

Supplementary Figure 11

PEG10

Tissue	Cancer Cell line	*SGCE/PEG10* Methylation (%)	CNV (total:minor allele)	rs13073 gDNA	rs13073 cDNA	rs13226637 gDNA	rs13226637 cDNA
Breast	MDA-MB-436	4	3:1	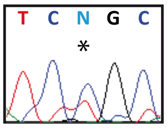	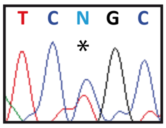		
	HCC-1954	55	5:2			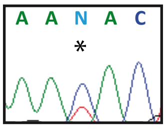	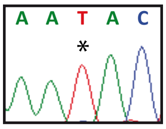
Colon	HCT-116	48	2:1			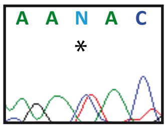	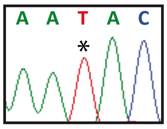
	NCI-H508	46	6:2			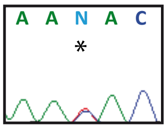	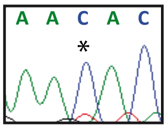
	SW-1417	53	4:2			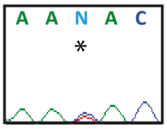	No expression
	COLO-205	72	3:1	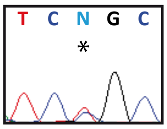	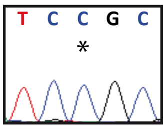		

Supplementary Fig. 11. Allelic expression analysis of 10 imprinted transcripts in cancer cell lines. The CNA status and average methylation for each DMR is shown alongside Sanger sequencing traces of highly informative polymorphisms in the *PEG10* transcript which were used for qualitative allelic expression analyses.

Supplementary Figure 12

H19

Tissue	Cancer Cell Line	*H19* ICR Methylation (%)	CNV (Total:minor allele)
Breast	HCC1143	27	3:1
	HCC1954	22	3:1
	MCF-10	No data	No data
	MDA-MB-231	55	3:1
Colon	LS-411N	46	4:2
	COLO-205	42	3:1
	LS-1034	47	4:2
Liver	HLE	13	3:1
Lung	A549	50	3:1
	MLI-H1299	11	3:1
	MLI-H522	46	3:1

rs2839704

gDNA cDNA

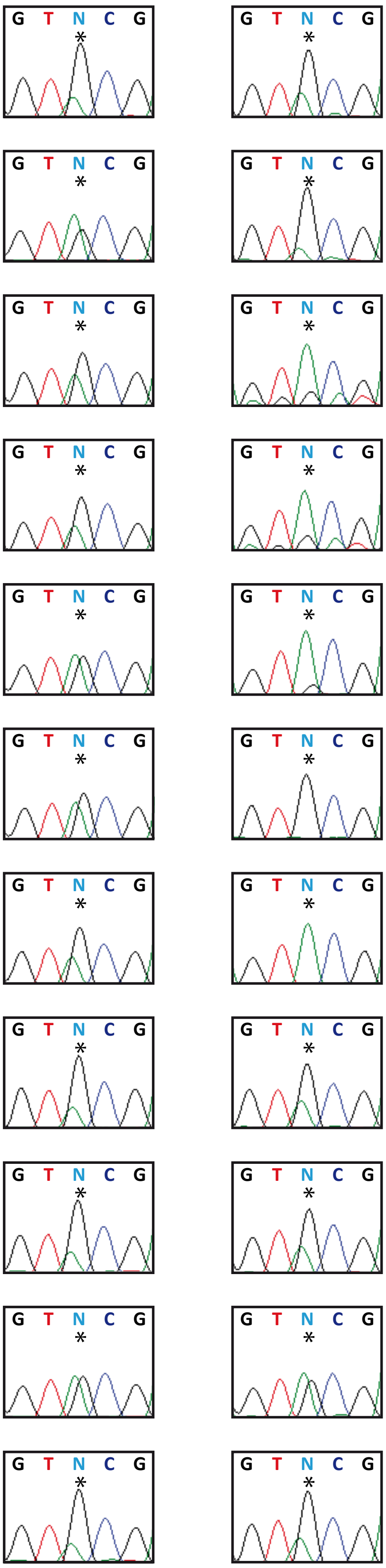

Supplementary Fig. 12. Allelic expression analysis of 10 imprinted transcripts in cancer cell lines. The CNA status and average methylation for each DMR is shown alongside Sanger sequencing traces of highly informative polymorphisms in the *H19* transcript which were used for qualitative allelic expression analyses.

Supplementary Figure 13

IGF2

Tissue	Cancer Cell Line	*IGF2* ICR Methylation (%)	CNV (Total:minor allele)	rs680 gDNA	rs680 cDNA
Breast	MDA-MB-231	55	3:1	G G N C C	G G C C C
	AU565	8	4:2	G G N C C	G G N C C
Colon	SW48	65	2:1	G G N C C	G G N C C
	NCI-H508	30	4:1	G G N C C	G G C C C
	HCT116	83	2:1	G G N C C	G G T C C
	COLO-205	42	3:1	G G N C C	G G N C C
Liver	HLE	13	3:1	G G N C C	G G N C C
	JHH-4	no data	no data	G G N C C	G G N C C
	JHH-2	no data	no data	G G N C C	G G N C C
Lung	A549	50	3:1	G G N C C	G G N C C
	NCI-H522	45	2:1	G G N C C	G G N C C
	NCI-H1975	45	3:1	G G N C C	G G N C C

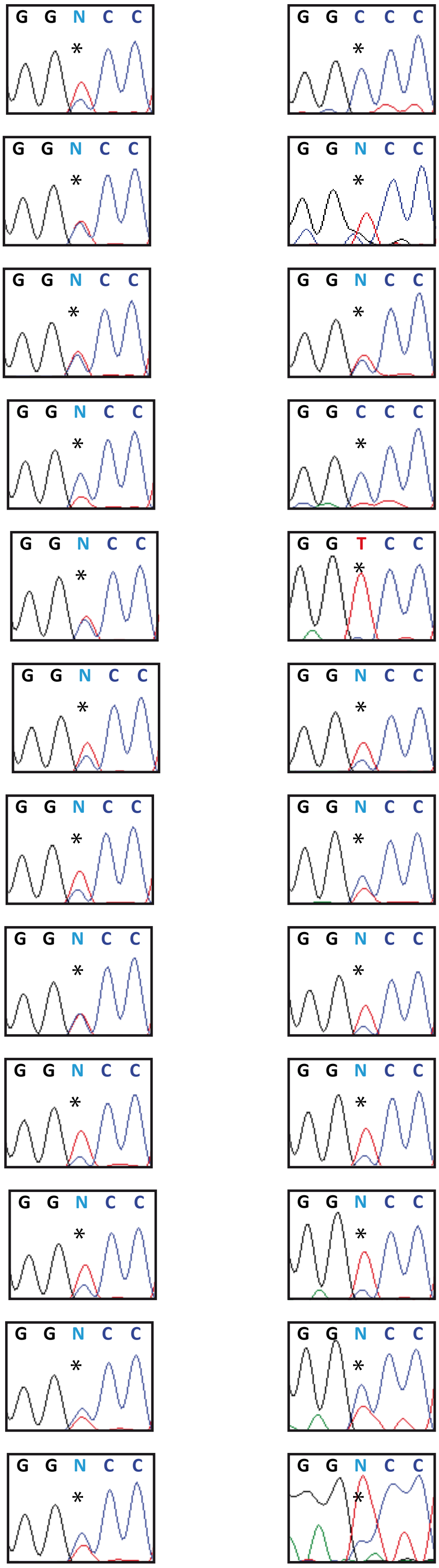

Supplementary Fig. 13. Allelic expression analysis of 10 imprinted transcripts in cancer cell lines. The CNA status and average methylation for each DMR is shown alongside Sanger sequencing traces of highly informative polymorphisms in the *IGF2* transcript which were used for qualitative allelic expression analyses.

Supplementary Figure 14

MEST

Tissue	Cancer Cell line	*MEST* DMR Methylation (%)	CNV (total:minor allele)	rs1050582 gDNA	rs1050582 cDNA
Breast	COLO-824	75	4:2	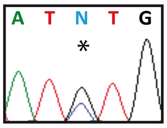	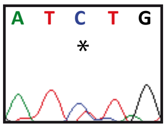
	MDA-MB-436	61	4:2	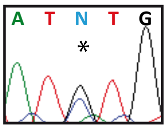	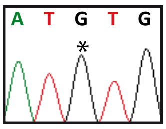
	MDA-MB-468	91	3:1	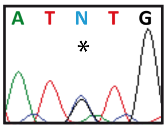	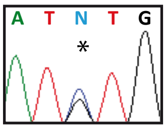
	AU565	87	7:2	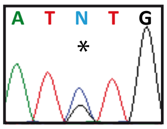	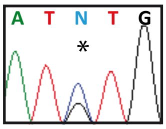
	HCC1937	80	4:2	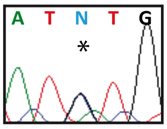	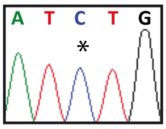
	BT-474	73	3:1	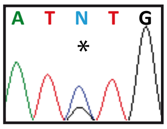	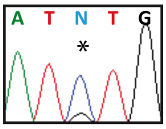
Colon	HT-29	93	4:2	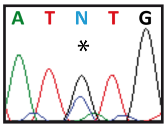	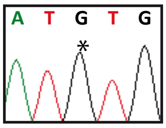
	SW620	93	3:1	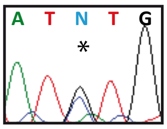	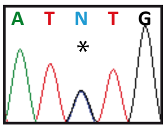
	SW1116	91	4:1	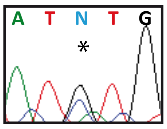	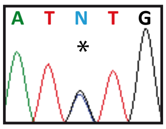
	HCT-15	91	2:1	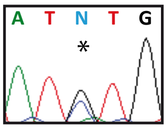	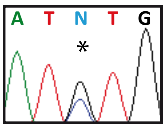
	LS-411N	92	5:2	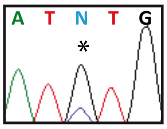	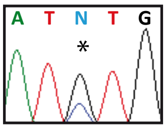
	GP5d	93	2:1	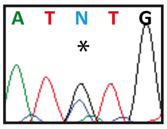	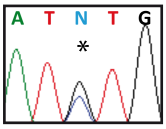
	HT55	91	4:2	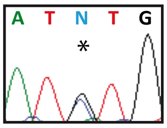	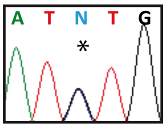
	HCT-116	92	2:1	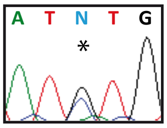	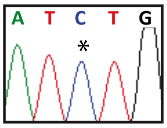

Supplementary Fig. 14. Allelic expression analysis of 10 imprinted transcripts in cancer cell lines. The CNA status and average methylation for each DMR is shown alongside Sanger sequencing traces of highly informative polymorphisms in the *IGF2* transcript which were used for qualitative allelic expression analyses.

Supplementary Figure 15

KCNQ1OT1

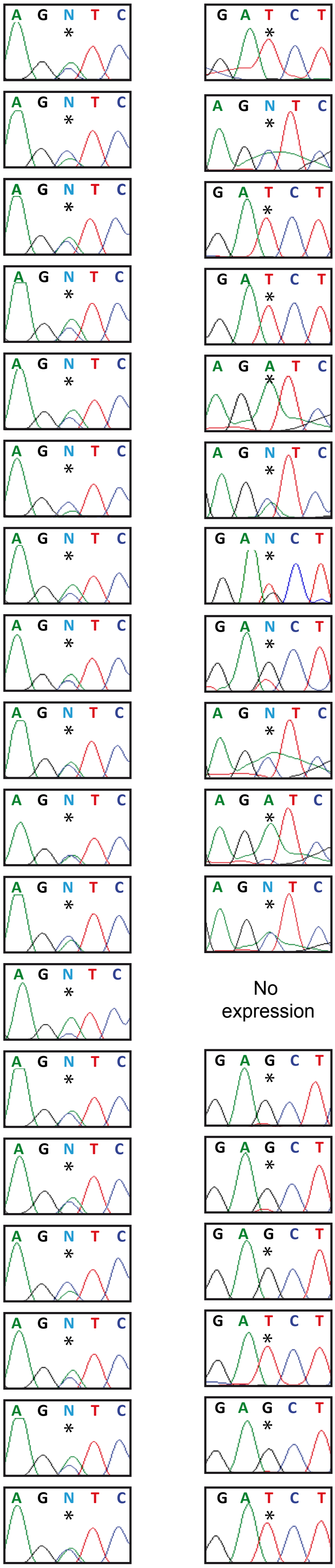

Tissue	Cancer Cell line	KvDMR Methylation (%)	CNV (total:minor allele)	rs231359 gDNA	rs231359 cDNA
Breast	AU565	25	4:2	A G N T C	G A T C T
	HCC1954	41	4:2	A G N T C	A G N T C
	MDA-MB-157	54	2:1	A G N T C	G A T C T
	Hs578T	28	2:1	A G N T C	G A T C T
	COLO-824	49	2:1	A G N T C	A G A T C
Colon	NCI-H508	66	4:1	A G N T C	A G N T C
	HCT-116	4	2:1	A G N T C	G A N C T
	HCT15	49	2:1	A G N T C	G A N C T
	COLO-205	54	3:1	A G N T C	A G N T C
	SW1417	55	4:2	A G N T C	A G A T C
	LS411N	59	4:2	A G N T C	A G N T C
Liver	HuCCT1		no data	A G N T C	No expression
	SNU-354		no data	A G N T C	G A G C T
Lung	NCI-H1048	53	2:1	A G N T C	G A G C T
	A549	41	3:1	A G N T C	G A G C T
	DMS-273	54	2:1	A G N T C	G A T C T
	NCI-H1993	51	4:1	A G N T C	G A G C T
	A522	56	3:1	A G N T C	G A T C T

Supplementary Fig. 15. Allelic expression analysis of 10 imprinted transcripts in cancer cell lines. The CNA status and average methylation for each DMR is shown alongside Sanger sequencing traces of highly informative polymorphisms in the *KCNQ1OT1* transcript which were used for qualitative allelic expression analyses.

Supplementary Figure 16

GLT2/MEG3

Tissue	Cancer Cell line	*MEG3* Methylation (%)	IG-DMR - Pyro Methylation (%)	CNV (total:minor allele)	rs11160608 gDNA	rs11160608 cDNA
Breast	MDA-MB-436	83	86	2:0	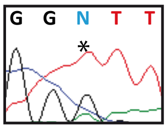	No expression
Breast	BT-474	86		3:1	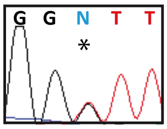	No expression
Breast	T47D	56	25	4:2	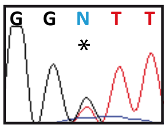	No expression
Breast	Hs578T	53		2:1	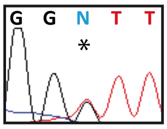	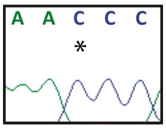
Colon	LS-411N	70	90	3:1	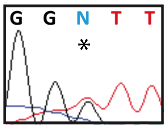	No expression
Colon	HCT-116	79	90	2:1	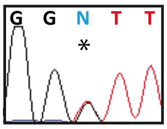	No expression
Colon	LoVo	75		2:1	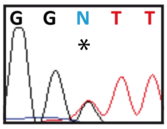	No expression
Colon	GP5d	82		2:1	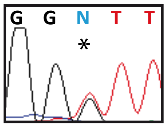	No expression
Colon	RKO	84		2:1	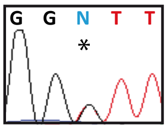	No expression
Colon	SW48	86	90	3:1	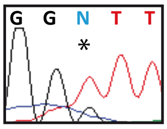	No expression
Colon	COLO-205	76		3:1	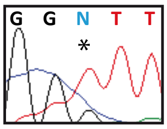	No expression
Colon	HT29	79	90	2:1	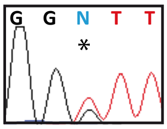	No expression
Colon	SW837	81	90	2:1	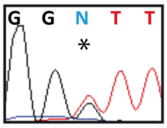	No expression

Supplementary Fig. 16. Allelic expression analysis of 10 imprinted transcripts in cancer cell lines. The CNA status and average methylation for each DMR is shown alongside Sanger sequencing traces of highly informative polymorphisms in the *MEG3* transcript which were used for qualitative allelic expression analyses.

Supplementary Figure 17

PEG3

Tissue	Cancer Cell line	*PEG3* Methylation (%)	CNV (total:minor allele)	rs4801386 gDNA	rs4801386 cDNA
Breast	MDA-MB-453	86	4:2	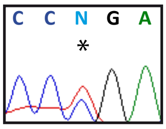	No expression
	MDA-MB-436	42	4:2	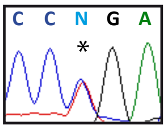	No expression
	HCC1143	74	4:2	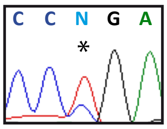	No expression
	HCC1937	57	7:3	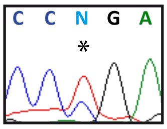	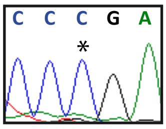
Colon	LS-411N	80	3:1	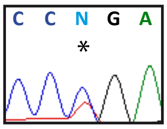	No expression
	HCT-116	73	2:1	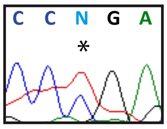	No expression
	HCT-15	79	2:1	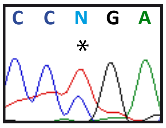	No expression
	SW48	85	2:1	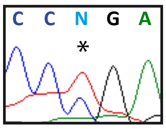	No expression
	HT-29	89	4:1	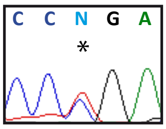	No expression
	NCI-H508	74	4:2	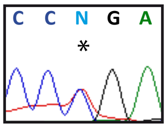	No expression
	HT-55	73	4:1	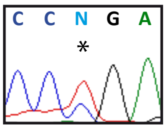	No expression

Supplementary Fig. 17. Allelic expression analysis of 10 imprinted transcripts in cancer cell lines. The CNA status and average methylation for each DMR is shown alongside Sanger sequencing traces of highly informative polymorphisms in the *PEG3* transcript which were used for qualitative allelic expression analyses.

Supplementary Figure 18

L3MBTL1

Tissue	Cancer Cell line	*L3MBTL1* Methylation (%)	CNV (total:minor allele)	rs1062943 gDNA	rs1062943 cDNA
Liver	Hep3B	no data	no data	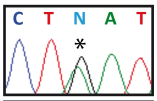	No expression
	AKNL	no data	no data	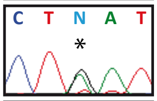	No expression
	SNU-354	no data	no data	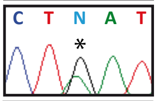	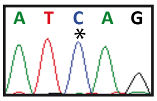
Colon	GP5d	0.52	2:1	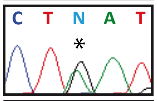	No expression
	HCT-116	0.42	2:1	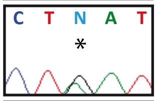	No expression
	HCT15	0.38	2:1	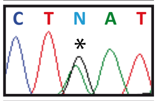	No expression
	SW48	0.46	2:1	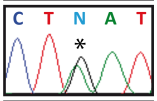	No expression
	COLO-205	0.39	5:2	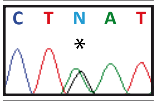	No expression
	HT29	0.42	5:2	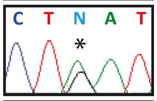	No expression
	LS-1034	0.39	5:2	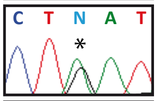	No expression
	SW620	0.44	3:1	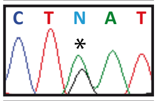	No expression
Breast	AU565	0.42	7:3	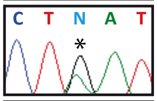	No expression
	HCC1143	0.44	4:2	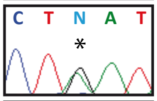	No expression
	UACC812	0.36	3:1	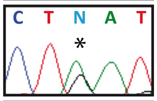	No expression
	MDA-MB-157	0.68	4:2	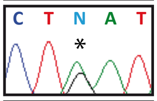	No expression
	MDA-MB-453	0.34	6:2	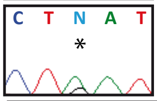	No expression
	MDA-MB-134 VI	no data	no data	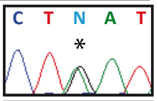	No expression
Lung	DMS-273	0.58	3:1	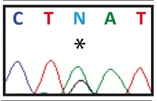	No expression
	NCI-H1975	0.51	4:2	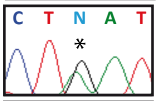	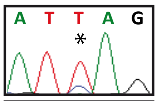
	NCI-H1993	0.39	6:3	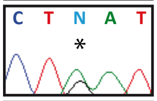	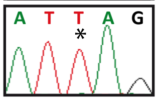
	NCI-H460	0.27	4:2	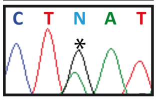	No expression

Supplementary Fig. 18. Allelic expression analysis of 10 imprinted transcripts in cancer cell lines. The CNA status and average methylation for each DMR is shown alongside Sanger sequencing traces of highly informative polymorphisms in the *L3MBTL1* transcript which were used for qualitative allelic expression analyses.

Supplementary Figure 19

NHP2L1

Tissue	Cancer Cell Line	*NHP2L1* Methylation (%)	CNV (Total:minor allele)	rs132806 gDNA	rs132806 cDNA
Breast	MDA-MB-453	35	5:2	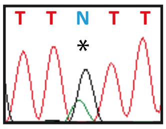	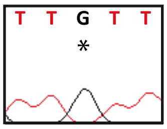
	Hs578T	53	4:2	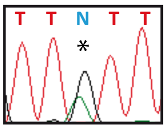	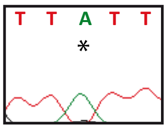
Colon	NCI-H508	47	4:2	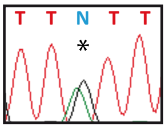	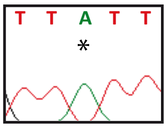
Liver	HLE	17	2:1	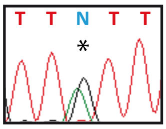	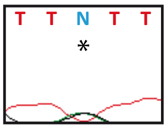
	Huh7	56	3:1	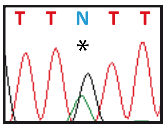	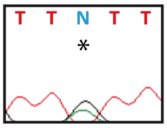
	SNU-368	no data	no data	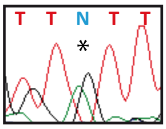	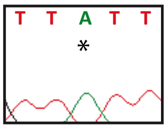
	SNU-354	no data	no data	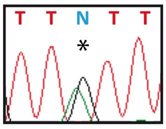	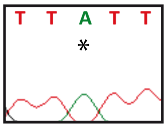
Lung	HCC1937	no data	no data	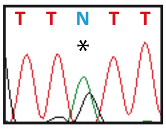	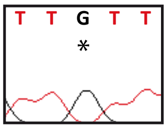
	NCI-H441	41	2:1	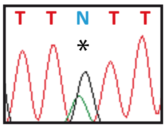	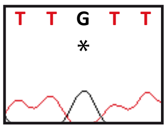

Supplementary Fig. 19. Allelic expression analysis of 10 imprinted transcripts in cancer cell lines. The CNA status and average methylation for each DMR is shown alongside Sanger sequencing traces of highly informative polymorphisms in the *NHP2L1* transcript which were used for qualitative allelic expression analyses.

Supplementary Figure 20

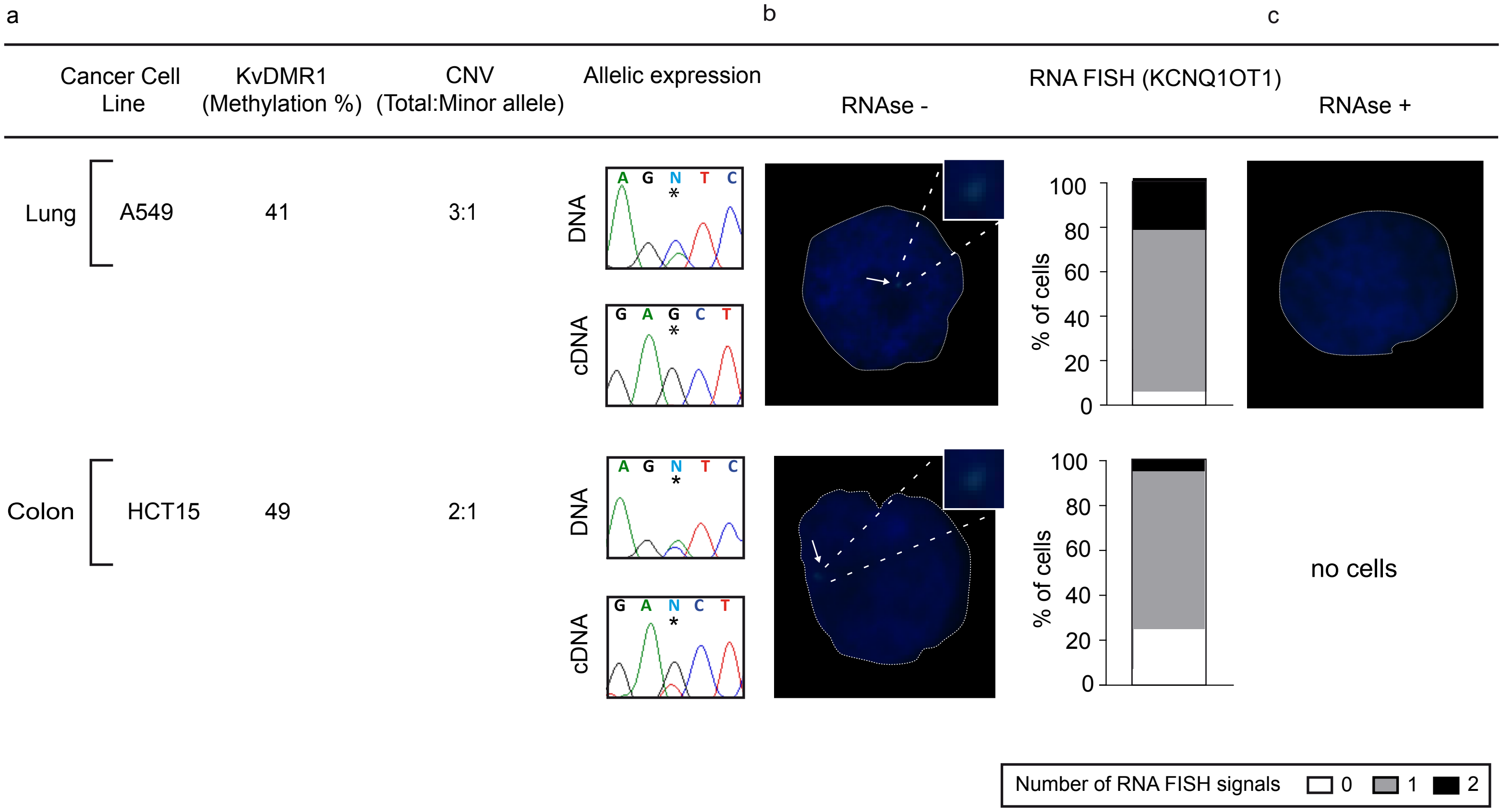

Supplementary Fig. 20. Allelic expression analysis of *KCNQ1OT1* in the lung cancer cell line A549 and the colon tumour cell lines HCT-15. (a) The average methylation of all Infinium HM450k probes mapping to the *Kv*DMR1 is shown alongside Sanger sequencing of rs231359 polymorphism in the *KCNQ1OT1* transcript in DNA and cDNA, respectively. (b) Representative RNA-FISH analysis of *KCNQ1OT1* lncRNA-coated territory (green signal, white arrows) of individual nuclei with inserts representing zoomed in immages of FISH signals. Nuclei were stained with DAPI. The quantification of *KCNQ1OT1* expression signals represented as stacked bar charts indicates the percentage of nuclei displaying the indicated number of expression foci. (c) *KCNQ1OT1* RNA-FISH performed on cells retreated with RNAse A.

Supplementary Figure 21

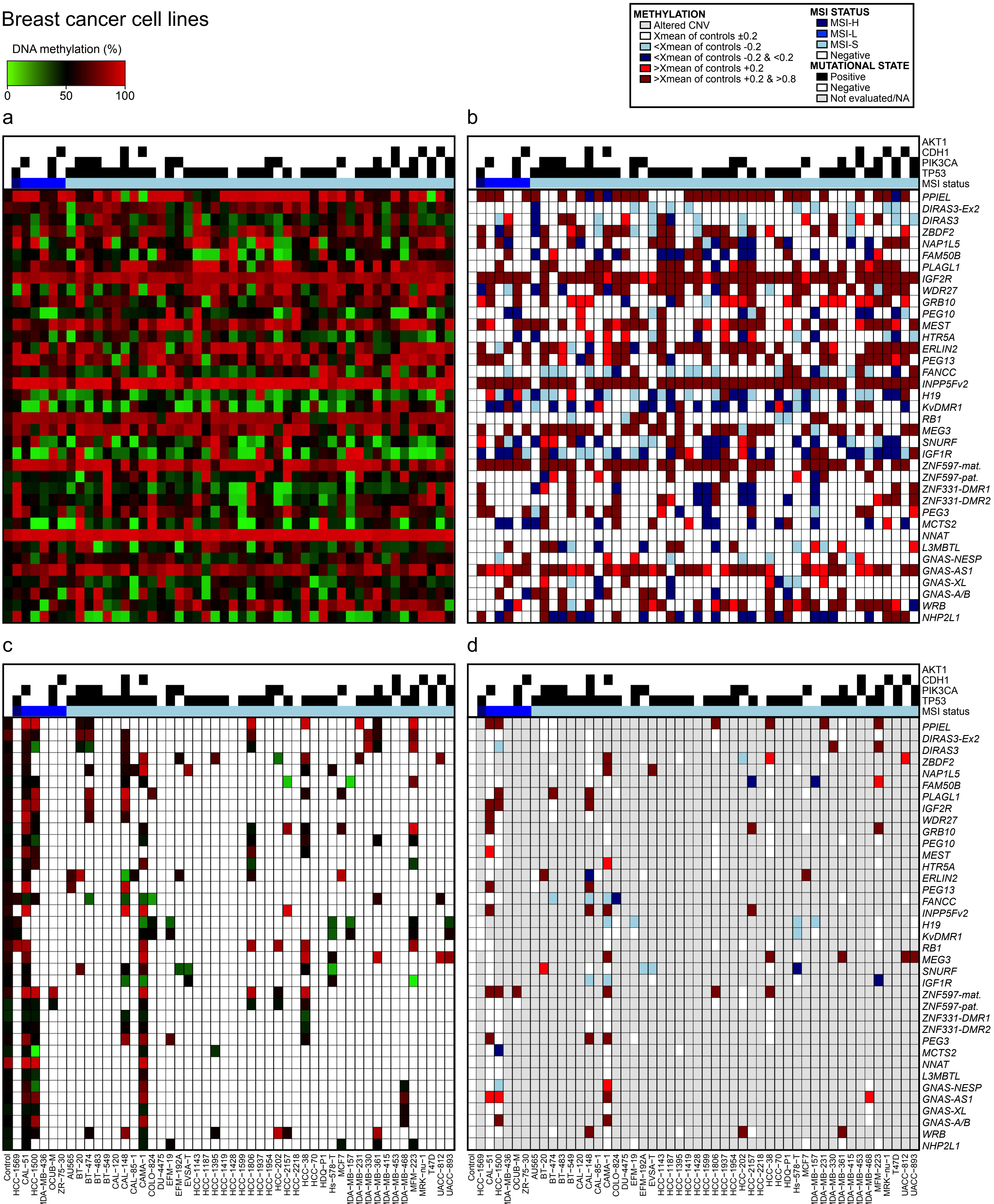

Supplementary Fig. 21. Imprinted DMR methylation profiles in breast cell lines compared to molecular and mutation status. For cell lines derived from breast tumours the imprinted DMR methylation profiles are compared to MSI status and the presence of *AKT1, CDH1, PIK3CA* and *TP53* mutations. (a) The average methylation of all HM450k probes mapping to individual imprinted DMR irrespectively of CNA status. (b) The methylation differences compared to tissue-matched controls. (c) The average methylation of HM450k probes mapping loci maintaining a normal 2:1 copy number. Data only presented for loci with normal copy-number. (d) The methylation differences for loci with 2:1 copy-number compared to control tissues. In this heatmap white data position represent no methylation change and light grey those regions with aberrant CNAs. The same figure panel outline is used for all other cancer cell line datasets. The methylation profile of the colorectal cancer cell lines were compared to MSI status and *TP53, MLH1, APC, KRAS, BRAF* and *PIK3CA* mutations; Hepatocellular carcinomas cell lines are compared to MSI status and *CTNNB1, TP53, AXIN1, JAK1* and *LRP1B* mutations; Lung cancer cell lines are compared to MSI status and *TP53,* and *PIK3CA* mutational status.

Supplementary Figure 22

Colon cancer cell lines

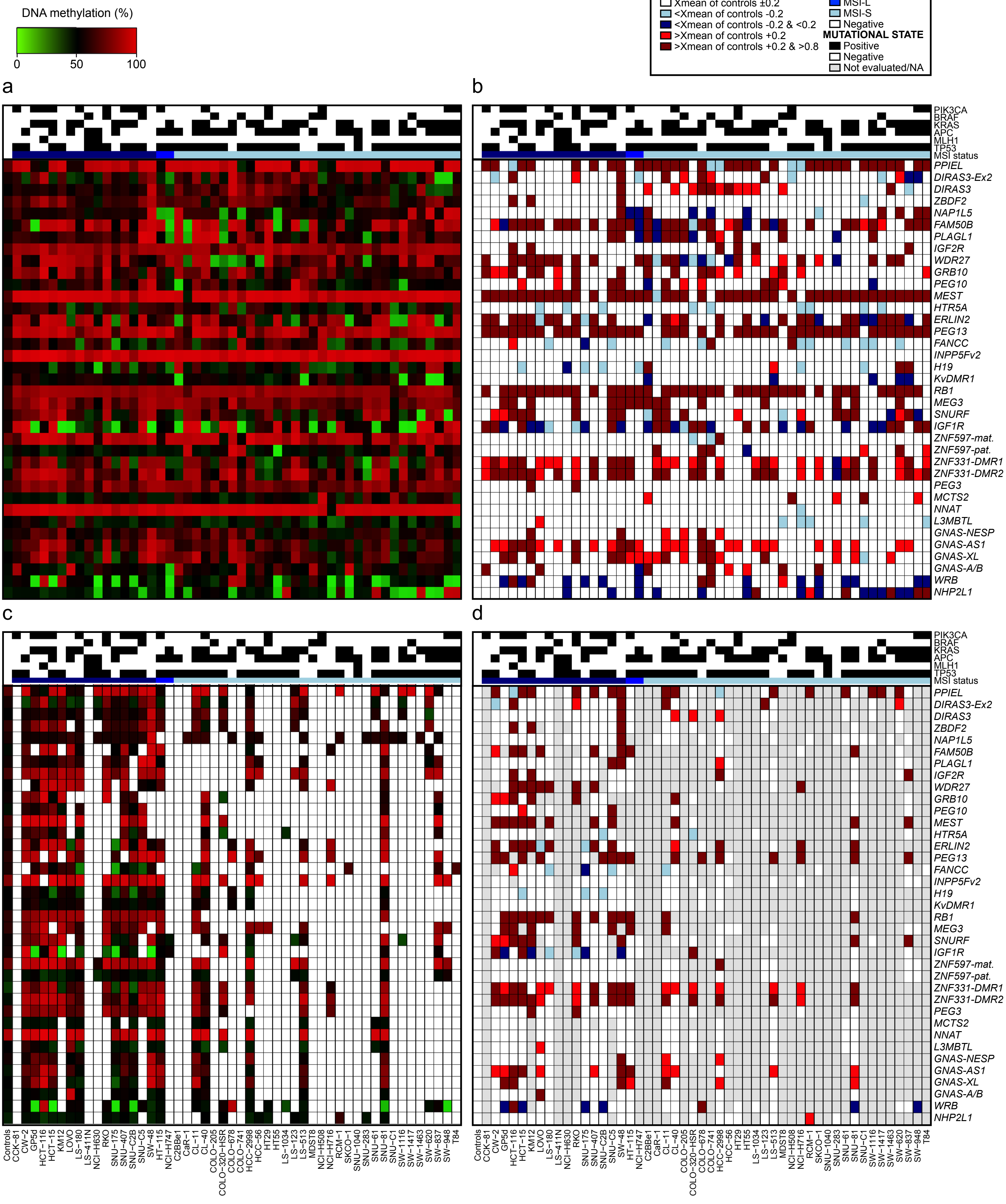

Supplementary Fig. 22. Imprinted DMR methylation profiles in colon cell lines compared to molecular and mutation status. For cell lines derived from breast tumours the imprinted DMR methylation profiles are compared to MSI status and the presence of *AKT1, CDH1, PIK3CA* and *TP53* mutations. (a) The average methylation of all HM450k probes mapping to individual imprinted DMR irrespectively of CNA status. (b) The methylation differences compared to tissue-matched controls. (c) The average methylation of HM450k probes mapping loci maintaining a normal 2:1 copy number. Data only presented for loci with normal copy-number. (d) The methylation differences for loci with 2:1 copy-number compared to control tissues. In this heatmap white data position represent no methylation change and light grey those regions with aberrant CNAs. The same figure panel outline is used for all other cancer cell line datasets. The methylation profile of the colorectal cancer cell lines were compared to MSI status and *TP53, MLH1, APC, KRAS, BRAF* and *PIK3CA* mutations; Hepatocellular carcinomas cell lines are compared to MSI status and *CTNNB1, TP53, AXIN1, JAK1* and *LRP1B* mutations; Lung cancer cell lines are compared to MSI status and *TP53,* and *PIK3CA* mutational status.

Supplementary Figure 23

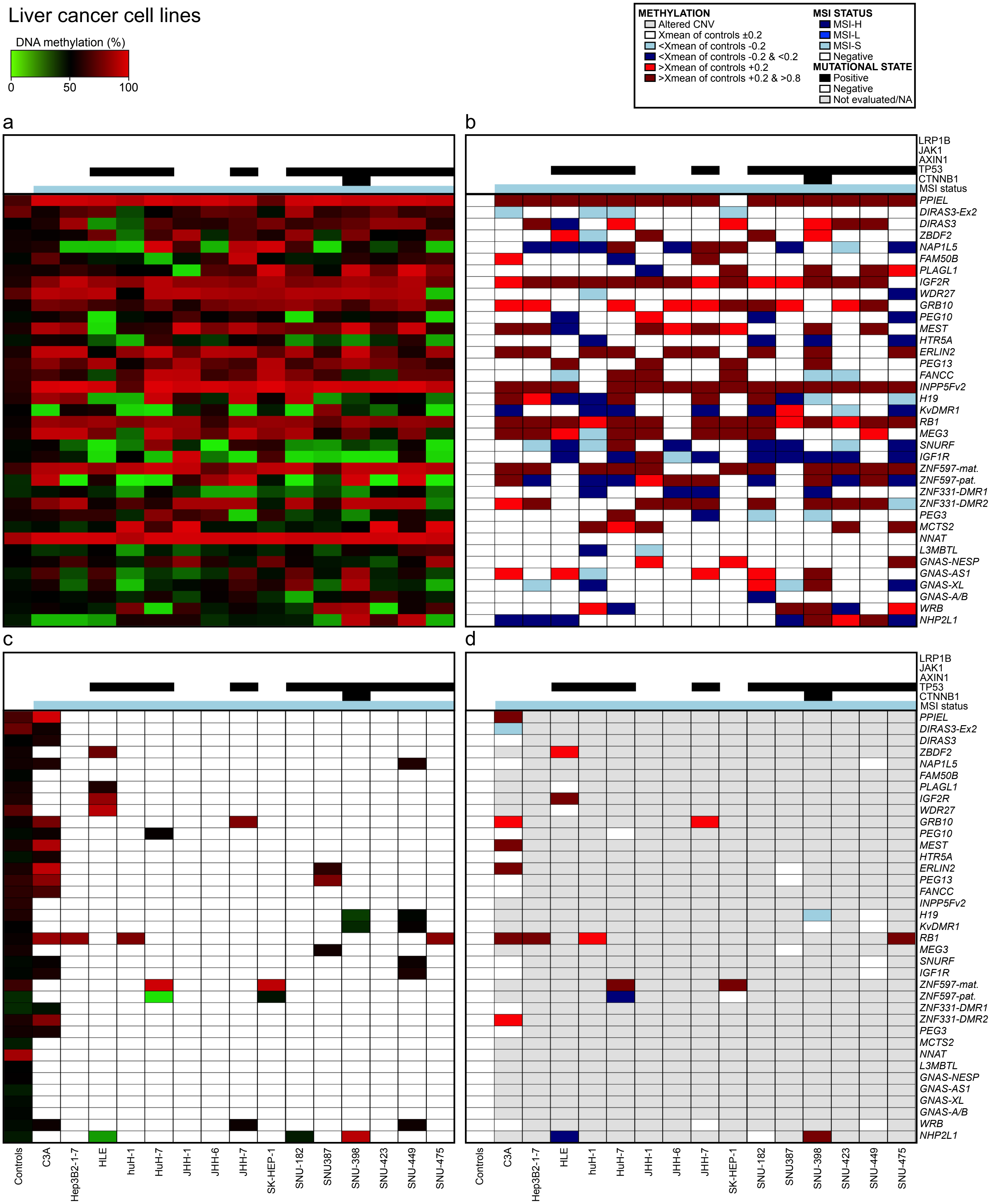

Supplementary Fig. 23. Imprinted DMR methylation profiles in liver cell lines compared to molecular and mutation status. For cell lines derived from breast tumours the imprinted DMR methylation profiles are compared to MSI status and the presence of *AKT1, CDH1, PIK3CA* and *TP53* mutations. (a) The average methylation of all HM450k probes mapping to individual imprinted DMR irrespectively of CNA status. (b) The methylation differences compared to tissue-matched controls. (c) The average methylation of HM450k probes mapping loci maintaining a normal 2:1 copy number. Data only presented for loci with normal copy-number. (d) The methylation differences for loci with 2:1 copy-number compared to control tissues. In this heatmap white data position represent no methylation change and light grey those regions with aberrant CNAs. The same figure panel outline is used for all other cancer cell line datasets. The methylation profile of the colorectal cancer cell lines were compared to MSI status and *TP53, MLH1, APC, KRAS, BRAF* and *PIK3CA* mutations; Hepatocellular carcinomas cell lines are compared to MSI status and *CTNNB1, TP53, AXIN1, JAK1* and *LRP1B* mutations; Lung cancer cell lines are compared to MSI status and *TP53,* and *PIK3CA* mutational status.

Supplementary Figure 24

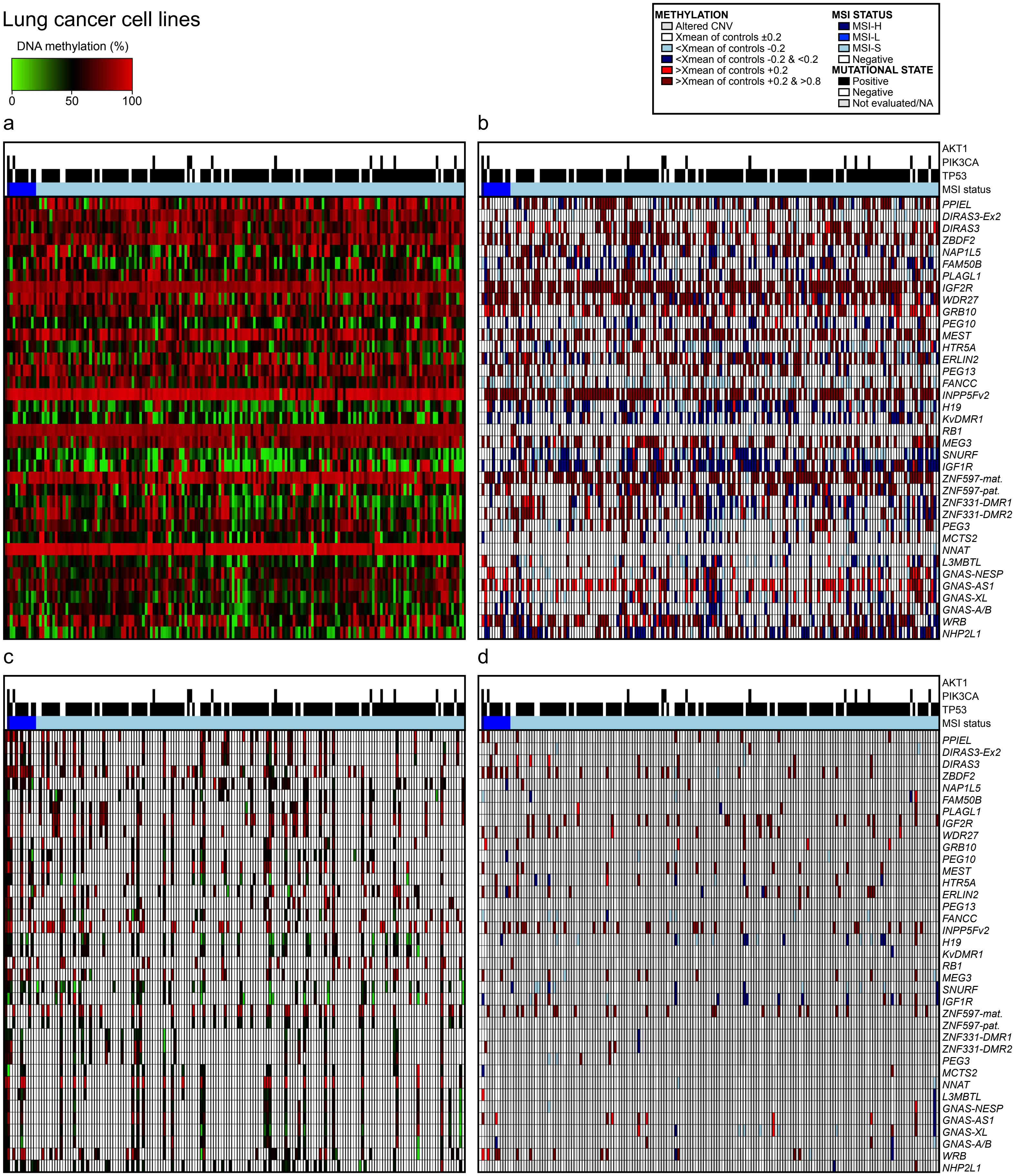

Supplementary Fig. 24. Imprinted DMR methylation profiles in lung cell lines compared to molecular and mutation status. For cell lines derived from breast tumours the imprinted DMR methylation profiles are compared to MSI status and the presence of *AKT1, CDH1, PIK3CA* and *TP53* mutations. (a) The average methylation of all HM450k probes mapping to individual imprinted DMR irrespectively of CNA status. (b) The methylation differences compared to tissue-matched controls. (c) The average methylation of HM450k probes mapping loci maintaining a normal 2:1 copy number. Data only presented for loci with normal copy-number. (d) The methylation differences for loci with 2:1 copy-number compared to control tissues. In this heatmap white data position represent no methylation change and light grey those regions with aberrant CNAs. The same figure panel outline is used for all other cancer cell line datasets. The methylation profile of the colorectal cancer cell lines were compared to MSI status and *TP53, MLH1, APC, KRAS, BRAF* and *PIK3CA* mutations; Hepatocellular carcinomas cell lines are compared to MSI status and *CTNNB1, TP53, AXIN1, JAK1* and *LRP1B* mutations; Lung cancer cell lines are compared to MSI status and *TP53,* and *PIK3CA* mutational status.

Supplementary Figure 25

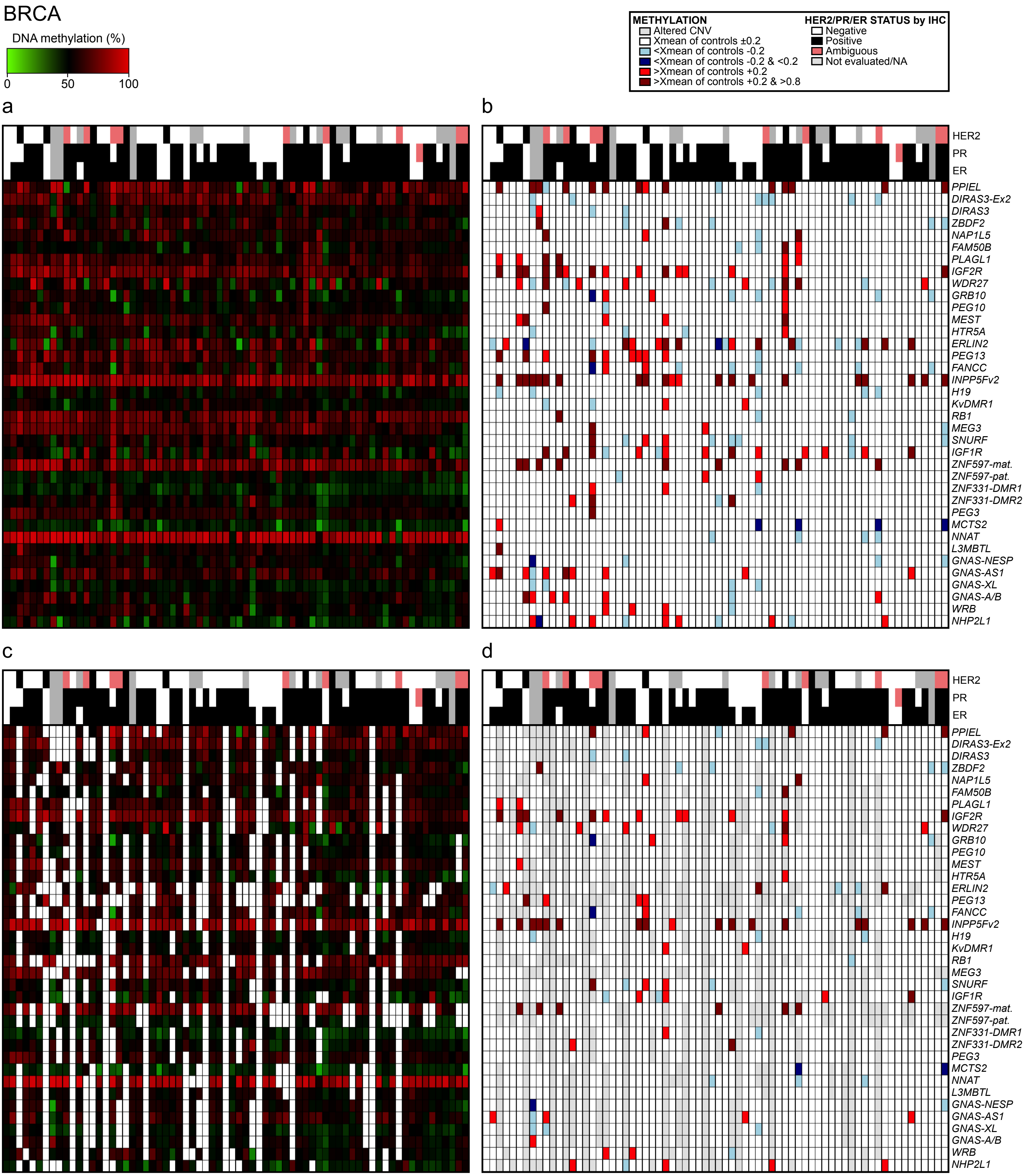

Supplementary Fig. 25. Imprinted DMR methylation profiles in TCGA datasets compared to molecular and clinical data. For the BRCA samples the imprinted DMR methylation profiles are compared to *HER2, PR* and *ER* expression status. (a) The average methylation of all HM450k probes mapping to individual imprinted DMR irrespectively of CNA status. (b) The methylation differences compared to adjacent control tissues. (c) The average methylation of HM450k probes mapping loci maintaining a normal 2:1 copy number. Data only presented for loci with normal 2:1 copy-number. (d) The methylation differences for loci with 2:1 copy-number compared to control tissues. In this heatmap white data position represent no methylation change and light grey those regions with aberrant CNAs. The same figure panel outline is used for all other TCGA tissue datasets assessed. COAD samples methylation profiles are compared to *KRAS* mutations, mismatch repair gene expression and MSI status; LIHC samples methylation profiles are compared to alcohol consumption, cirrhosis and hepatitis B and C status (hepatitis history, presence of surface antigens and positive serology); LUAD samples methylation profiles are compared to smoking status and the presence of *EML4* translocations*, EGFR* and *KRAS* mutations.

Supplementary Figure 26

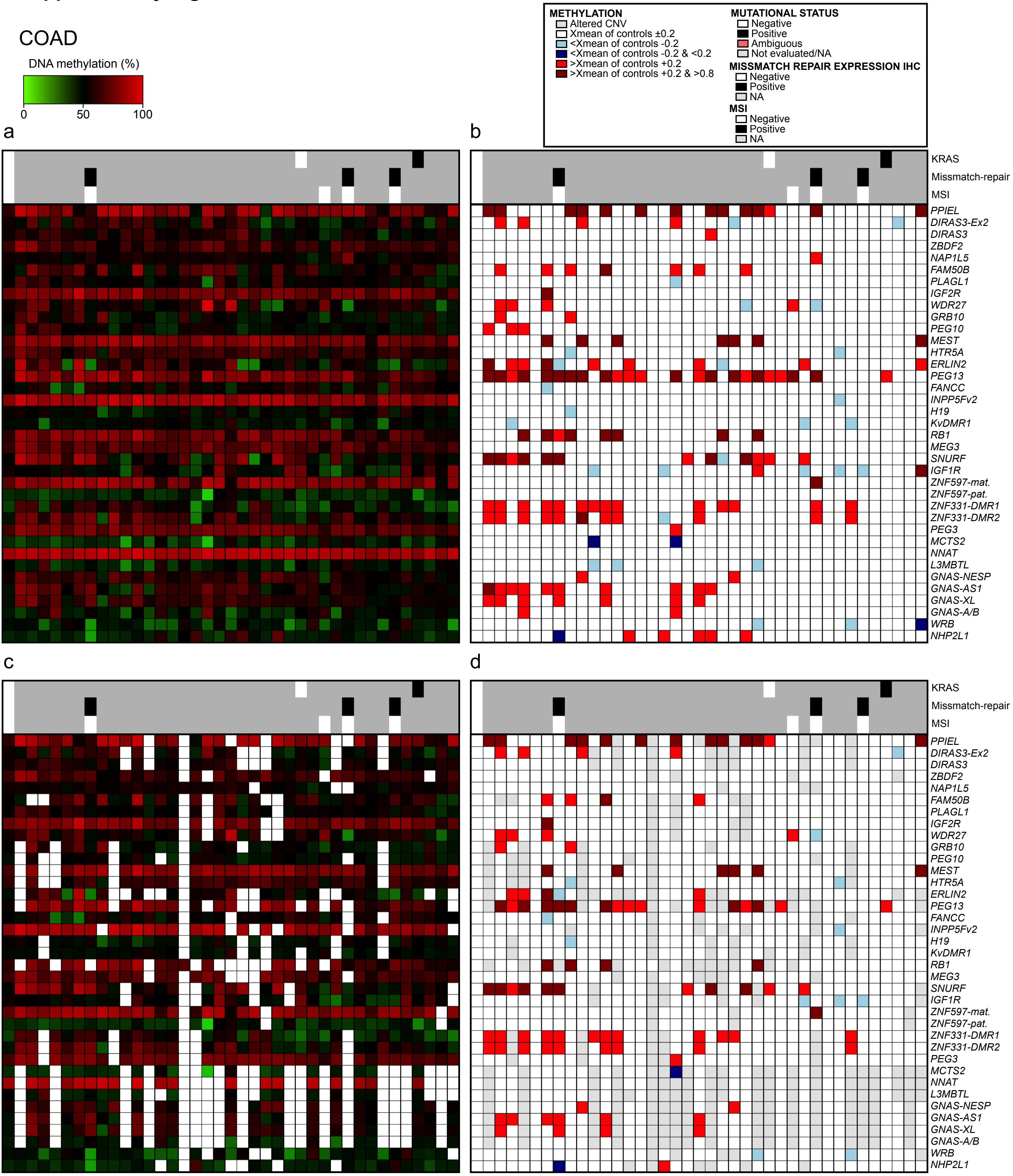

Supplementary Fig. 26. Imprinted DMR methylation profiles in TCGA datasets compared to molecular and clinical data. For the BRCA samples the imprinted DMR methylation profiles are compared to *HER2, PR* and *ER* expression status. (a) The average methylation of all HM450k probes mapping to individual imprinted DMR irrespectively of CNA status. (b) The methylation differences compared to adjacent control tissues. (c) The average methylation of HM450k probes mapping loci maintaining a normal 2:1 copy number. Data only presented for loci with normal 2:1 copy-number. (d) The methylation differences for loci with 2:1 copy-number compared to control tissues. In this heatmap white data position represent no methylation change and light grey those regions with aberrant CNAs. The same figure panel outline is used for all other TCGA tissue datasets assessed. COAD samples methylation profiles are compared to *KRAS* mutations, mismatch repair gene expression and MSI status; LIHC samples methylation profiles are compared to alcohol consumption, cirrhosis and hepatitis B and C status (hepatitis history, presence of surface antigens and positive serology); LUAD samples methylation profiles are compared to smoking status and the presence of *EML4* translocations, *EGFR* and *KRAS* mutations.

Supplementary Figure 27

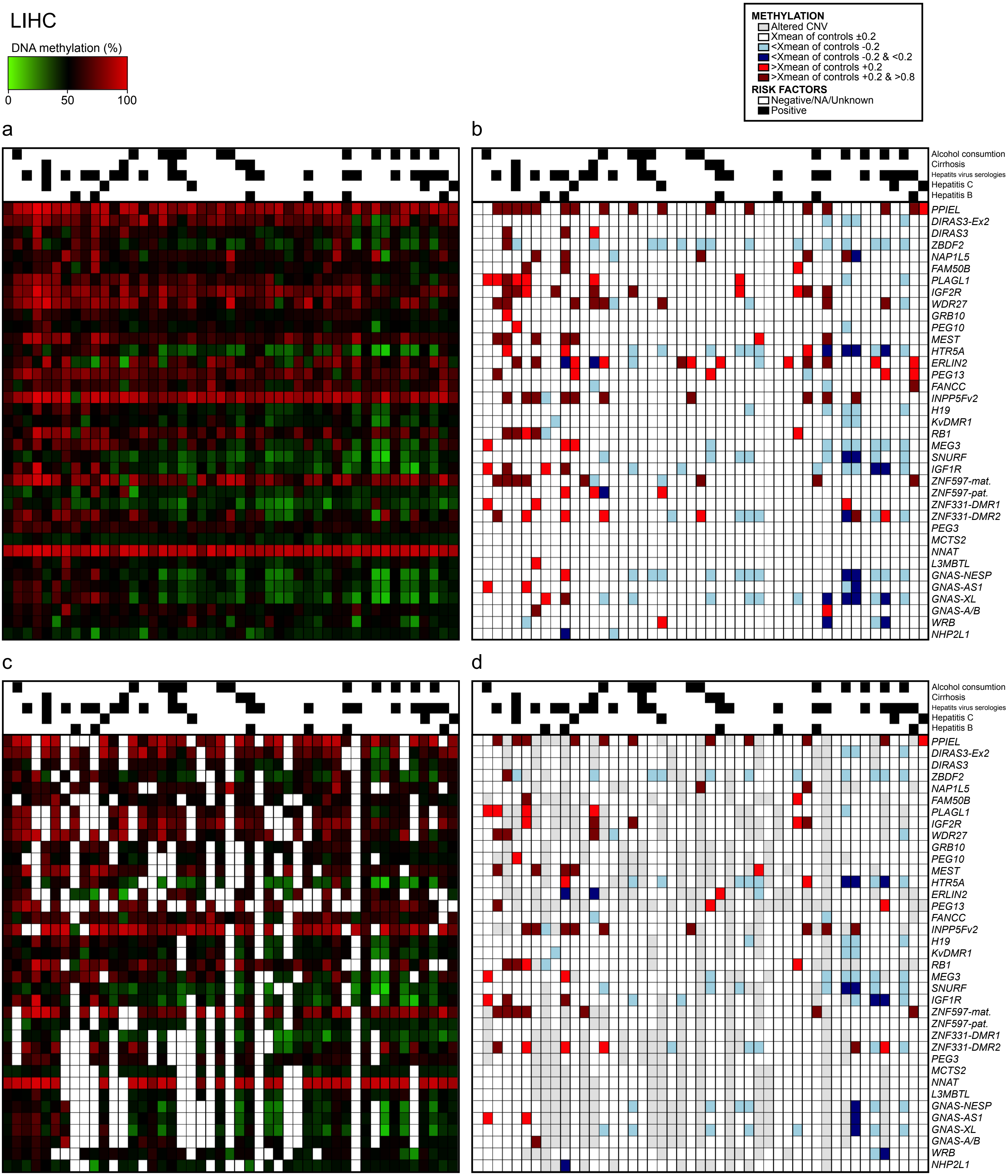

Supplementary Fig. 27. Imprinted DMR methylation profiles in TCGA datasets compared to molecular and clinical data. For the BRCA samples the imprinted DMR methylation profiles are compared to *HER2, PR* and *ER* expression status. (a) The average methylation of all HM450k probes mapping to individual imprinted DMR irrespectively of CNA status. (b) The methylation differences compared to adjacent control tissues. (c) The average methylation of HM450k probes mapping loci maintaining a normal 2:1 copy number. Data only presented for loci with normal 2:1 copy-number. (d) The methylation differences for loci with 2:1 copy-number compared to control tissues. In this heatmap white data position represent no methylation change and light grey those regions with aberrant CNAs. The same figure panel outline is used for all other TCGA tissue datasets assessed. COAD samples methylation profiles are compared to *KRAS* mutations, mismatch repair gene expression and MSI status; LIHC samples methylation profiles are compared to alcohol consumption, cirrhosis and hepatitis B and C status (hepatitis history, presence of surface antigens and positive serology); LUAD samples methylation profiles are compared to smoking status and the presence of *EML4* translocations, *EGFR* and *KRAS* mutations.

Supplementary Figure 28

LUAD

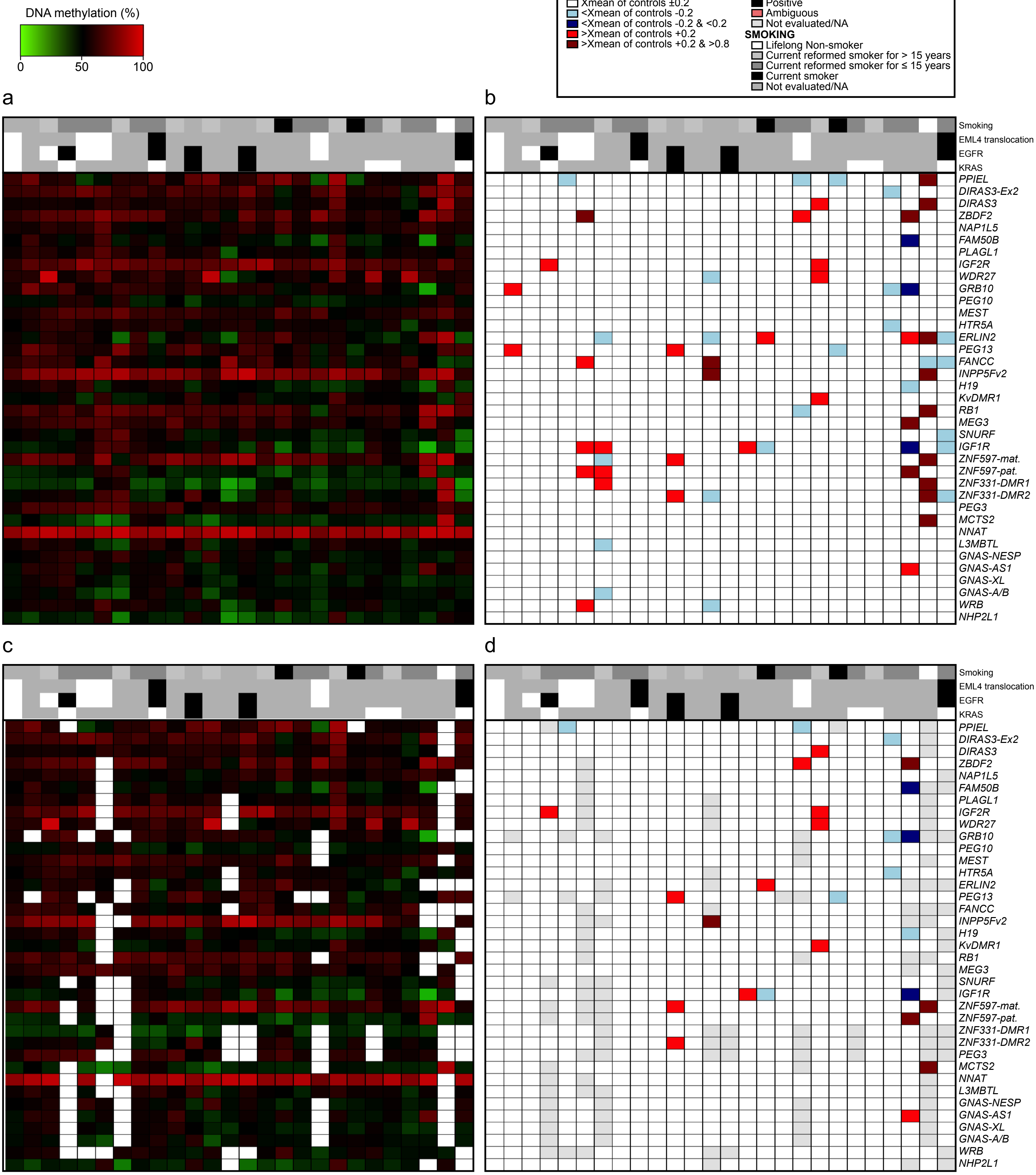

Supplementary Fig. 28. Imprinted DMR methylation profiles in TCGA datasets compared to molecular and clinical data. For the BRCA samples the imprinted DMR methylation profiles are compared to *HER2*, *PR* and *ER* expression status. (a) The average methylation of all HM450k probes mapping to individual imprinted DMR irrespectively of CNA status. (b) The methylation differences compared to adjacent control tissues. (c) The average methylation of HM450k probes mapping loci maintaining a normal 2:1 copy number. Data only presented for loci with normal 2:1 copy-number. (d) The methylation differences for loci with 2:1 copy-number compared to control tissues. In this heatmap white data position represent no methylation change and light grey those regions with aberrant CNAs. The same figure panel outline is used for all other TCGA tissue datasets assessed. COAD samples methylation profiles are compared to *KRAS* mutations, mismatch repair gene expression and MSI status; LIHC samples methylation profiles are compared to alcohol consumption, cirrhosis and hepatitis B and C status (hepatitis history, presence of surface antigens and positive serology); LUAD samples methylation profiles are compared to smoking status and the presence of *EML4* translocations*, EGFR* and *KRAS* mutations.
